# Group-based cardiac telerehabilitation interventions and health outcomes in coronary patients: A scoping review

**DOI:** 10.1177/02692155231202855

**Published:** 2023-09-21

**Authors:** Renuka J Jacobsson, Anne Oikarinen, Jenni Krogell, Päivi Kankkunen

**Affiliations:** 1 Department of Nursing Science, Faculty of Health Sciences, 205537 University of Eastern Finland, Kuopio, Finland; 2 Research Unit of Health Sciences and Technology, Faculty of Medicine, University of Oulu, Oulu, Finland

**Keywords:** Coronary disease, group-based, cardiac telerehabilitation, scoping review

## Abstract

**Objective:**

To explore the extent and type of evidence in relation to group-based cardiac telerehabilitation interventions and health outcomes in coronary artery disease patients.

**Data sources:**

A literature search was conducted in August 2022 and July 2023 in databases including PubMed, CINAHL, Scopus and PsycINFO. The search process followed the scoping review methodology guided by the Joanna Briggs Institute for scoping reviews.

**Methods:**

The inclusion criteria were a peer-reviewed journal article published in English between 1 January 2017 and 15 August 2022 and updated to cover until 15 July 2023 concerning group-based cardiac telerehabilitation in adult coronary artery disease patients. All group-based cardiac telerehabilitation interventions and health outcome types were charted and summarized.

**Results:**

The researcher screened a total of 2089 articles, of which 22 were retained with a total of 1596 participants. Group-based cardiac telerehabilitation interventions were particularly useful for patients with multi-faceted technological applications and social support. The patients received guidance regarding cardiovascular disease risk factors. Physical fitness, psychological complaints and quality of life were often measured outcomes in the included studies.

**Conclusions:**

This scoping review indicates the success of various rehabilitation interventions utilizing different technologies for coronary patients. Coronary patients were guided in making lifestyle changes, and positive findings were observed in the health outcomes measured after the telerehabilitation intervention. The findings of this review can provide valuable guidance for developing and evaluating sustainable group-based cardiac telerehabilitation programs that aim to benefit coronary patients.

## Introduction

Coronary artery disease causes the most morbidity and deaths in the world.^1,2^ After an acute cardiac event, the prevention of new cardiac events is essential and reduces suffering.^3^ This secondary prevention includes the methods used to prevent the progression of coronary disease in a patient who is already ill.^4^ Evidence-based prevention includes medication optimization, risk factor management and lifestyle changes.^5,6^ The recommendation for cardiac rehabilitation spread to different countries, putting emphasis on the provision of rehabilitation guidance and secondary prevention in the treatment process of cardiac patients.^7^ Telerehabilitation is the purposeful use of applications that utilize digital technology in rehabilitation.^8^ Rehabilitation groups serve various purposes, such as educating patients, practicing skills and strategies, and providing opportunities for peer support.^9^

The goal of rehabilitation is disease management^10^ which means the improvement of the patient's physical, psychological, social and overall life situation by means of rehabilitation. Death statistics have traditionally been used as outcome variables,^11^ but more recently, changes in risk factors and quality of life have also been included as outcome variables.^10^ While the effects of telerehabilitation interventions on cardiac patients have been studied earlier,^12–14^ there is still a lack of a comprehensive overview of interventions and the different outcomes of telerehabilitation groups. The findings of this review are expected to provide a broad view of evidence-based information available on group-based cardiac telerehabilitation. The aim of this scoping review was to explore the extent and type of evidence related to group-based cardiac telerehabilitation interventions and health outcomes in coronary artery disease patients. The research questions that guided this scoping review were: (1) What kinds of group-based cardiac telerehabilitation interventions have been used in coronary patients? and (2) What health outcome types have been identified in coronary patients participating in group-based cardiac telerehabilitation interventions?

## Methods

We used a scoping review methodology recommended by the Joanna Briggs Institute scoping reviews.^15^ This involved carrying out five steps for scoping reviews.^16^ The identification of the research question (step 1) was presented in the introduction. The identification of potential studies, inclusion/exclusion review and data charting (steps 2–4) are summarized in this section. This is followed by findings/recommendations (step 5) presented in the results and discussion section.

The inclusion criteria applied to the database search were defined according to the aim of the review and the review questions. A literature search was conducted in August 2022 and updated in July 2023 in the PubMed, CINAHL, Scopus, and PsycINFO databases. The authors developed a valid research strategy and comprehensive list of search terms in consultation with a university librarian. The databases were thoroughly searched for topics relevant to group-based cardiac telerehabilitation using the following general terms: “cardiac,” “coronary,” “cardiovascular,” “rehabilitation,” “virtual,” “mobile,” “mobile apps,” “smartphone,” “telenursing,” “telemedicine,” and “ehealth.” The limits applied to the database search were publication in English and studies published from 1 January 2017 to 15 August 2022 and, in the updated search, from 1 August 2022 to 15 July 2023 (Supplemental Material Table 1). This yielded 2089 articles retrieved from the data sources described previously.

The selection criteria were defined according to the inclusion criteria which followed the Joanna Briggs Institute Manual and the PCC model, wherein P stands for population, C for concept and C for context.^15^ In this review, the population of interest comprises studies concerning coronary patients aged over 18 years in rehabilitation groups. The concepts examined in this review are group-based cardiac telerehabilitation interventions and health outcomes. Group-based cardiac telerehabilitation interventions are programs used to increase participants’ levels of self-care, potentially improving their lifestyles. Group-based cardiac telerehabilitation refers to e-technology (telephone, mobile phone, computer, tablet computer and wearables) and includes the use of applications, providing real-time/asynchronous feedback as well as systems that facilitate remote communication with healthcare professionals and their support.^11^ The context examined in this review involves remote/digital environments in any healthcare setting where cardiac rehabilitation is provided. The present review includes qualitative, quantitative and mixed-method studies and full-text empirical articles with an abstract available. Systematic reviews, epidemiological studies, and study protocols/designs were excluded (Table 1).

**Table 1.** Inclusion and exclusion criteria.

Inclusion criteria	Exclusion criteria
English language Peer-reviewed Group-based cardiac telerehabilitation for patients with coronary disease Studies of coronary patients over the age of 18 Reported about at least one group-based cardiac telerehabilitation intervention or outcomes of group-based cardiac telerehabilitation in coronary patients	Studies whose results included patients with chronic diseases or stroke Studies in which technology use did not include rehabilitation Studies that concerned cardiac rehabilitation without technology use Studies in which cardiac telerehabilitation did not include a group-based intervention Systematic reviews, epidemiological studies, study protocols and study designs

The review followed the PRISMA-ScR (preferred reporting items for systematic reviews and meta-analyses for scoping reviews) guidelines.^17^ The database search identified a total of 2089 documents. After removing duplicates, two authors (RJJ and JK) independently screened 1475 articles identified in the literature search based on their titles/abstracts. This was followed by a full-text assessment of 44 articles, which involved assessing each article regarding whether it fulfilled the inclusion or exclusion criteria. Those articles that met the inclusion criteria were selected for inclusion in the study. Any disagreements were discussed between the two researchers (RJJ and JK) or a third researcher was consulted (AO) until a consensus was reached. The final selection of relevant articles comprised 22 articles (Supplemental Material Figure 1). A data charting table was developed by the author.^1^Information was recorded as follows: author(s), year of publication, study location, study population and sample, aim, method, intervention type, intervention setting, outcome measures, follow-up time, and health outcome(s) (Appendix 1). Covidence® software was used for screening and data extraction.

## Results

### Characteristics of the studies

A total of *N*  =  22 articles were included in this review.^18–39^ The studies varied in research designs, including 11 randomized controlled trials,^18–20,23,26,27,30–33,35^ five pilot studies,^21,24,25,28,36^ two observational studies,^29,34^ one case study,^22^ one qualitative study,^37^ one mixed-method study,^38^ and one cross-sectional study.^39^ The studies had been conducted in different locations: 11 of them in Europe,^18–20,22,28,31,33–36,39^ six in Asia,^23,24,29,30,32,37^ three in Australia,^25–27^ and two in North America.^21,38^ The target population of 21 of the studies was coronary artery disease patients with heart failure and in one study coronary artery disease patients and the patients’ partners.^22^ All patients participated in the studies after percutaneous coronary revascularization or coronary artery bypass graft. The study samples ranged from 8^21^ to 335^29^ coronary artery disease patients.

### Intervention types

The participants took part in education sessions delivered by expert health professionals. In the 22 studies, the health professionals were mostly nurses^22,30–32,34,37–39^ and physiotherapists,^20,26,27,30,35,39^ and a minority were cardiologists and doctors,^20,26,27^ clinicians,^26^ cardiovascular disease specialists, dietitians or technical staff.^21,34,35,38^ Out of the 22 studies, 19 described technology-based coaching and feedback provided by healthcare professionals.^18,21,23–35,37–39^ In the most recently published studies, motivational messages and feedback had been included and were produced by technology by coding these to the used electronic platform.^32,35,37^ Only six of the studies clearly described group interactions and social support interactions.^22,24,26,30,35,38^

The duration of the cardiac telerehabilitation interventions ranged from 6 weeks^32^ to 12 weeks.^18,19,21,22,33^ A minority of the studies described the process of developing intervention content. The behavioral theory had been used in two of the studies,^25,31^ learning theory in one study,^22^ and the chronic disease model in one study.^27^ Many interventions were delivered in a healthcare setting and tracked remotely. The patients participated in the interventions in their homes, receiving a home-based exercise intervention with telemonitoring guidance.^18,20,24,28–31,33^ Telerehabilitation was particularly useful when patients were not able to access clinical facilities^29^ or were motivated by the easy access and reduced travel time enabled by the solutions.^24,27^

In all the studies, the interventions were computer- or tablet-based. Patients participating in the group-based cardiac telerehabilitation were required to have their own, relevant technological equipment with Internet access (computer, telephone, and mobile connection).^18–39^ A variety of other devices and applications were also used in the included studies (Supplemental Material Table 3). Out of the 22 studies, a chat feature was primarily utilized as a means of delivering the intervention in 15 studies,^21,22,24–27,29–32,34,35,37–39^ telemonitoring,^18,20,24,28–30,32–37,39^ and a virtual platform^21–25,28,30–32,34,35,37–39^ were used in 14 studies, other mobile connections in 12 studies^18,19,23,24,28,29,33–37,39^ and video conferencing in nine studies.^21,24,26,27,30,34,36,38,39^ Using video conferencing at patients’ homes enabled clinicians to observe the participants, provide them with feedback and facilitate peer-support,^24,27,38^ and this method was used for an exercise and education intervention delivered by healthcare professionals.^25–27^

Of the 22 studies, five involved physical exercise testing performed using remote telemonitoring devices (e.g. smart watch, digital step counter, blood pressure monitor, and fitness tracker).^18,19,34,35,39^ Of the 22 studies, four utilized remote patient heart rate monitoring.^18,26,33,39^ Most of the studies included some elements of self-management resources. These elements aimed to boost behavioral risk factor management in coronary artery disease patients.^25^ The behaviors mainly included physical activity and diet.^21,25,34,38,39^ Only a minority of studies addressed or described risk factors related to smoking or hypertension management^20,32,34,37^ or other issues most relevant to cardiac rehabilitation (sexuality and heart disease, heart medications).^21^ In 11 of the 22 studies, the intervention participants were encouraged to set goals and take action to modify their behavioral risk factors focused on healthy lifestyle changes.^18,20,23,25,26,31,32,34,35,37–39^ In two of the 22 studies, returning to work had been set as the goal.^25,35^ Online platforms were used to empower patients to make progress toward their goal^34,37,39^ and aimed at supporting patients in planning their coping.^21^

The used chat feature was commonly text-based and synchronous, using two-way audio–visual communication that enabled interactions between all parties.^26^ Many cardiac rehabilitation participants enjoyed the group interactions and social support enabled by such solutions.^26^ Participants shared their experiences and pursued their goals together in an online group.^32^ Elderly patients were conservative about using online communication.^24^ Patients were encouraged to post their questions on the forum to get responses from the nurse and their peers.^32^ Healthcare staff also contacted individual participants if a need to discuss their progress was detected.^31^ Four studies described the use of telecommunication specifically for the purpose of guiding the participants on how to use technology in rehabilitation. This included activities such as sharing instructional information and operating technical devices.^19,21,31,35^

### Measured health outcome types

In this context, health outcome types refer to changes in the coronary artery patients’ health status and to patient-reported outcomes. Health outcomes were collected at baseline in all the studies as well as at post-intervention measurement points from 6 weeks^32^ to 4^33^ years after the intervention (Table 2). Out of the 22 studies, the health outcome measurement point was 6 weeks in five studies^20,23–25,30^ but in most of the studies, this was set at 12 weeks.^18,19,21–23,26,27,29,31,33^ In total, 11 studies used longer follow-up times. These included four studies with measurement points set at 6 months,^26,27,30,34^ five studies with measurement points at 1 year,^18,19,29,31,33^ one study with a measurement point at 2 years^29^ and one study at 4 years.^33^

**Table 2.** Summary of the health outcomes of the group-based cardiac telerehabilitation interventions in the included studies.

Outcome(s)	Direction	Follow-up time	Studies
General health	No change	12 weeks	Avila et al.^18^ (2018)
	BMI no change	12 weeks and 1 year 6 and 12 weeks 12 weeks	Batalik et al.^19^ (2021) Su and Yu^32^ (2021) Duan et al.^23^ (2018)
	Better health habits Muscle strength: no change Mood and balance: no change	12 weeks	Brewer et al.^21^ (2017) Hwang et al.^27^ (2017)
	Positive behavioral changes General lifestyle profile improved Smoking status: no change	6 and 12 weeks	Su and Yu^32^ (2021)
	Lifestyle improved	3, 6 and 12 months 8 weeks	Spindler et al.^31^ (2019) Brewer et al.^38^ (2023)
Physical fitness	Peak VO_2_ P was larger	12 weeks and 1 year	Avila et al.^18^ (2018)
	No change	12 weeks	Batalik et al.^19^ (2021) Duan et al.^23^ (2018)
	No change 6-minute walk test: no change Muscle strength: no change	12 weeks, 1 and 4 years 12 weeks and 6 months	Brouwers et al.^33^ (2022) Hwang et al.^26^ (2017)
	Mood and balance improved	12 weeks	Hwang et al.^27^ (2017)
	6-minute walk test improved 6-minute walk test no change	2, 4 and 6 months 12 months 8 weeks	Peng et al.^30^ (2018) Lahtio et al.^35^ (2023) Giggins et al.^36^ (2023)
	No change in functional, balance and muscle strength	6 weeks	Su and Yu^32^ (2021)
	Improved exercise capacity Improved physical activity	24–42-month period 8 weeks 6 months 8 weeks	Ma et al.^29^ (2021) Calvo-López et al.^39^ (2023) Gibson et al.^34^ (2023) Giggins et al.^36^ (2023)
Social complaints	Better online forum use Better part of community	12 weeks	Dinesen et al.^22^ (2019)
	Support from family, other participants and clinicians	12 weeks	Hwang et al.^27^ (2017)
	Offered social support by professionals and peers	12 weeks	Su et al.^37^ (2023)
Psychological complaints	Sense of individuality improved Sense of autonomy improved	12 weeks	Dinesen et al.^22^ (2019)
	Sense of competence improved	12-weeks	Hwang et al.^27^ (2017)
	Motivation improved	2, 4 and 6 months	Peng et al.^30^ (2018)
	Anxiety and depression: no change Depression: no change	12 weeks 8 weeks	Duan et al.^23^ (2018) Lin et al.^24^ (2018)
	Anxiety and depression reduced	6 months 8 weeks	Gibson et al.^34^ (2023) Calvo-López et al.^39^ (2023)
	Psychological status: no change	24–42-month period	Ma et al.^29^ (2021)
Quality of life	No change	12 weeks	Avila et al.^18^ (2018)
	No change	12 weeks and 1 year 8 weeks	Batalik et al.^19^ (2021) Duan et al.^23^ (2018)
	Improved	12 weeks 12 weeks and 1 year	Bravo-Escobar et al.^20^ (2017) Brouwers et al.^33^ (2022)
	Improved	2, 4 and 6 months 12 weeks	Peng et al.^30^ (2018) Su and Yu^32^ (2021)
	Improved	8 weeks 8 weeks	Duan et al.^23^ (2018) Calvo-López et al.^39^ (2023)
Health knowledge	Improved	12 weeks 12 weeks	Brewer et al.^21^ (2017) Su et al.^37^ (2023)
	Satisfied with visiting the self-learning platform	8 weeks	Lin et al.^24^ (2018)
Adherence to treatment	Self-management improved	8 weeks	Higgins et al.^25^ (2017)
	Self-efficacy improved	12 weeks	Su and Yu^32^ (2021)
	Use of medication: no change	8 weeks	Bravo-Escobar et al.^20^ (2017)
	Attendance in rehabilitation improved	12 weeks and 6 months	Hwang et al.^26^ (2017)

Common strategies used to promote a healthy lifestyle included promoting behavioral changes.^21,23,32,34,38,39^ Among those with a healthy lifestyle at the start of the intervention, all the patients in the cardiac telerehabilitation group continued to maintain their healthy lifestyle throughout the treatment.^23^ Early recovery prior to the return to work increased motivation for change and supported sustaining long-term self-management.^25^ The return to work and its significance were not measured in other studies. In 22 of the studies, nine measured traditional risk factors and showed no significant differences in anthropometric characteristics or traditional cardiovascular risk factors,^18,19,23,29,32,35,36,38,39^ although clinically relevant trends were noted in some studies.^38,39^ Several studies describe that patients have many risk factors.^38^ In one study, the majority were smokers, and about half of the participants reached the targeted blood pressure.^29^ One study reported improvement in diet,^34^ but most of the studies reported no significant improvements in the patients’ dietary or smoking status.^32^ Body mass index associated with risk factors was calculated using the formula BMI  =  weight/height in five of the 22 studies. After a 6–12-week period^19,23,32,34,36^ and a 1-year follow-up,^19^ the participants’ BMIs continued to be similar. Only one study showed reductions in mean weight in a 6-month follow-up.^38^ A 42-month follow-up showed better control of risk factors regarding LDL-C and systolic blood pressure.^29^ The use of prescription medications was measured in one study.^20^

In total, nine studies included physical fitness tests. Physical fitness was tested based on a functional capacity test,^27,34–36^ oxygen uptake,^18^ pVO_2_,^19,33^ the number of steps^32^ and a 6-minute walk test.^27,30,35^ In total, in eight of the nine studies, the participants’ physical fitness increased. Oxygen uptake at the first and second ventilatory thresholds increased more in the 12-week follow-up.^18^ Functional exercise capacity increased 4 months after the post-test in an 8-week cardiac telerehabilitation program.^30^ The average peak of pVO_2_ was higher after 1-year follow-up.^19^ One study showed improvement in the number of steps per day.^32^ Muscle strength, mood and balance were improved.^27^ After 1-year follow-up, a cardiac telerehabilitation had improved the participants’ physical activity, but a long-term follow-up 4-years after the cardiac telerehabilitation showed that the intervention had not prevented a relapse in physical activity.^33^ However, it conveys that in all the studies analyzed, there was no association or correlation found between telerehabilitation and improvement in physical activity.^27,32,33^

Social complaints were measured in a total of four studies. Group-based cardiac telerehabilitation improved social support when patients received support from healthcare professionals^20,22,27,37^ and peer support from other participants.^22,25,27,37^ Healthcare workers coached patients to return to their everyday lives.^22^ Support from family was improved during cardiac telerehabilitation, with participants spending more time with their families and carrying out the same exercises.^26^ Opportunities to interact with other cardiac patients online were clearly desired by patients. The rehabilitation programs provided emotional support through shared experiences.^20,25^ The online sessions promoted group cohesion and a sense of universality. The participants acknowledged that they were communicating with real people, which shortened the psychological distance between group members despite their physical distance.^24^

Psychological complaints were measured in 11 of the 22 studies.^22,23,25,27,31–34,37–39^ Anxiety and depression were measured in six studies, and most of them found no differences in the scores for anxiety and depression^23,24,29,30,34,38^ except one study.^34^ The participants’ sense of individuality, autonomy, capability and motivation improved.^22^ In one study, improvement was only found in subscales between the 1- and 4-year follow-up in the control group but not in the telerehabilitation intervention group.^33^ An initial increase was observed in autonomous motivation but this positive change in motivation did not last over time.^31^ High motivation to use the self-learning platform and participate in video conferences indicated that a group-based cardiac telerehabilitation program is feasible and acceptable for the psychosocial rehabilitation of patients with coronary artery disease.^24,37^

In total, seven of the 22 studies measured health-related quality of life. None of these studies found significant differences in the quality of life between control and intervention groups.^18,19,23,30–32,35^ Quality of life seems to improve over time in a 4-month to 1-year follow-up.^30,32^ Apparently, an exercise training program improved participants’ quality of life after the start of the cardiac telerehabilitation.^30^

In total, five of the 22 studies included a health knowledge measurement.^21,25,31,37,38^ In one study, most of the participants reported that the program had improved their health knowledge and helped them maintain better health habits.^21^ The patients believed that the online cardiac rehabilitation program would be more appropriate for patients with lower health literacy than those participating in the rehabilitation.^25^ While satisfaction with the rehabilitation was measured in the studies, health knowledge improvement was not measured in connection with rehabilitation.^24^

Only three of the 22 studies measured self-management and self-efficacy.^25,32,37^ A 12-week empowerment-based telerehabilitation program was found to improve patients’ self-efficacy and was seen to support self-management in patients ready and willing to implement it.^32^ Self-efficacy emerged as an increased activity in carrying out lifestyle changes and coping plans.^25,35^ A total of two studies measured motivation.^24,27^ The participants were highly motivated and satisfied with using the self-learning platform and the video conference feature.^24^ Improved motivation influenced health outcomes and access to care.^27^

## Discussion

The aim of this scoping review was to examine the different types of interventions and health outcomes targeted at coronary patients. This review may clarify definitions and provide contextual information on telerehabilitation phenomena. To our knowledge, this is the first scoping review of group-based cardiac telerehabilitation. Most of the earlier studies focus on individual telerehabilitation without a group-based context.

There is limited literature describing the use of group-based chats or video conferencing as a part of cardiac rehabilitation. Most interventions included online meetings between groups and healthcare professionals and these meetings enabled support and communication. The most recent studies showed an increase in technology-based motivational messages in addition to interpersonal communication. Using artificial intelligence-based communication for support has not been studied much in the context of rehabilitation, and there is no clear evidence regarding the current understanding of patients’ views and preparedness to use artificial intelligence in their practices.^40^ A positive finding in this study is the support and guidance provided by healthcare professionals to patients. Although physiotherapists and nurses play a significant role and accompany patients during rehabilitation, this aspect was not clearly described in the studies. The role of cardiac nurses is very important in overall cardiac rehabilitation leading to an improvement in the patient's well-being and recovery.^41^ In the future, the role of nurses in interventions should be described in greater depth and with more precision. Our findings support the assumption that group-based social support can be used in rehabilitation groups to provide opportunities for peer support^9^; indeed, geographically isolated patients were found to benefit the most from the communication included in the rehabilitation.^24^ Telemedicine was particularly useful when patients were unable to access clinic facilities^29^ or were motivated by easy access and reduced travel time.^24,27^

This scoping review demonstrated that the interventions utilized different digital tools and platforms. Various applications and digital devices were used significantly differently throughout the interventions. We suggest that telerehabilitation interventions can be implemented with a wide variety of devices and communication applications. The elderly population exhibited reduced levels of participation in communication.^24^ This finding was in line with previous studies on telerehabilitation, which observed challenges related to the use of technology, especially among older users.^42^ To conclude, the digital devices available to elderly coronary patients and their knowledge of using them need to be reviewed. As a result, there is a clear demand for further investigation of telerehabilitation programs targeted at elderly coronary patients. In the future, it would be relevant to investigate clear instructions for patient-friendly and patient-centered digital and communication tools, and standardized remote methods are essential for clinical practice.

Long-term outcomes were not addressed in the included studies, which typically contained follow-up times of 6, 8 and 12 weeks. This was also identified by a previous review on telerehabilitation, in which the included studies did not address any long-term outcomes.^42^ Further studies will provide insight into the long-term impacts of group-based cardiac telerehabilitation. The studies did not measure the most useful time to start rehabilitation after a cardiac event. This is significant to internal psychological resources (high intention and good self-regulation capability) as a central variable and should especially be considered in health promotion aimed at cardiac patients.^23^

In this review, we were primarily interested in the types of interventions and outcomes reported in the studies. The achieved outcomes and results showed improvement in patients’ general health, physical capacity, psychological well-being, quality of life and self-effectiveness. A personalized approach to risk stratification and invasive procedures combined with optimal medical therapy improved finding an effective solution for the prevention of new cardiac events in the patients.^3^ Patients had an interest in optimizing their medication, and while this was a topic of guidance and education, its effect was not measured in the included studies. As a result, future research should pay attention to adherence to medication. Smoking cessation, as an outcome variable, was also almost absent in the studies.^32^ However, smoking cessation is recommended as an important goal in cardiac rehabilitation because smoking is a modifiable risk factor that should be pursued in every secondary cardiac rehabilitation program. International and European guidelines recommend that coronary artery disease patients should be encouraged to stop smoking permanently.^13^ In the future, it would be relevant to investigate how to support patients in smoking cessation and what methods can be used in technology-based rehabilitation.

Sample sizes in the included studies were small; in most of the studies, the sample comprised less than 50 patients. In most of the studies, all patients had been recruited from a single clinic. Based on the recommendation for normally distributed outcomes, the relative gain in precision of the pooled standard deviation (SDp) is less than 10% with a total sample size of 70 and 5% with a pilot sample size of 60. If the event rate in an intervention group needs to be estimated by a pilot, a total of 60–100 patients is required in the pilot trial, and for a primary outcome, a total of at least 120 subjects (60 in each group) may be required.^43^ More reliable and significant results will be obtained in the future when larger sample sizes are used.

Most of the included studies were from Europe and Asia, and several of them were from Australia. Only one of the European studies was from the Nordic countries,^35^ even though the prevalence of heart disease is high in the Nordic countries. Most of the European coronary patients have a less than optimal management of LDL-C and lifestyle changes are needed.^5,44^ In a previous study, it was discovered that the majority of coronary patients did not have their blood pressure, low-density lipoprotein cholesterol and glucose targets under control.^44^ Cardiovascular disorders are largely preventable, which provides grounds for optimism that the spread of favorable mortality trends in the high-income countries of Western Europe will continue, as inequalities in prevention and treatment are diminishing.^45^ Our review demonstrates that providing groups with remote support through technology can lead to positive lifestyle changes among coronary patients. Results from the European Society of Cardiology EUROASPIRE V registry indicate that cardiovascular prevention requires introducing a modern preventive cardiology program delivered by interdisciplinary teams of healthcare professionals addressing all aspects of lifestyle and risk factor management to reduce the risk of recurrent cardiovascular events.^44^ For this reason, this scoping review showed that the included interventions likely resulted in improvements in the quality of life, physical activity and psychological well-being of coronary patients. However, while the patients received guidance in controlling risk factors, the risk factors were not sufficiently and comprehensively measured at the follow-ups.

This scoping review highlights the success of various rehabilitation interventions utilizing different technologies for coronary patients. The coronary artery disease patients were guided and supported in making lifestyle changes, but changes in cardiovascular disease risk factors, lifestyle or medication were often not measured in the included studies. The conclusion is that there is a need for a comprehensive assessment of the outcomes of the intervention on a full range of secondary prevention measures such as cardiovascular disease risk factor modifications, psychosocial complaints, adherence to treatment, rehospitalizations and adherence to medication along with lifestyle changes. Group-based cardiac telerehabilitation should be delivered by multidisciplinary teams addressing all relevant aspects and the means of managing risk factors to reduce the risk of recurrent cardiovascular events. Telerehabilitation should be promoted by health policy and healthcare management due to its potential for reducing inequality. By enabling participation in rehabilitation whatever their clinical facilities and regardless of geographical distance, telerehabilitation can help bridge the gap in healthcare access and promote equity among patients. In clinical practice, technological tools can be harnessed to guide, monitor and actively involve patients in their treatment process. In the future, it is suggested that interventions should be designed to promote long-term adherence to healthy lifestyles and sustainable changes in coronary patients.

**Clinical messages**

- Based on the results, it can be suggested that nurses and other healthcare professionals can effectively utilize group-based telerehabilitation models to assist cardiac patients in establishing and achieving healthier lifestyles.
- Group-based cardiac telerehabilitation enables peer support from other participants, group interaction and social support.
- Different technological interventions can be used in cardiac rehabilitation for patients who are geographically isolated, and clinical facilities for rehabilitation are limited.

## Supplemental Material

sj-docx-1-cre-10.1177_02692155231202855 - Supplemental material for Group-based cardiac telerehabilitation interventions and health outcomes in coronary patients: A scoping review Click here for additional data file. Supplemental material, sj-docx-1-cre-10.1177_02692155231202855 for Group-based cardiac telerehabilitation interventions and health outcomes in coronary patients: A scoping review by Renuka J Jacobsson, Anne Oikarinen, Jenni Krogell and Päivi Kankkunen in Clinical Rehabilitation

sj-docx-2-cre-10.1177_02692155231202855 - Supplemental material for Group-based cardiac telerehabilitation interventions and health outcomes in coronary patients: A scoping review Click here for additional data file. Supplemental material, sj-docx-2-cre-10.1177_02692155231202855 for Group-based cardiac telerehabilitation interventions and health outcomes in coronary patients: A scoping review by Renuka J Jacobsson, Anne Oikarinen, Jenni Krogell and Päivi Kankkunen in Clinical Rehabilitation

sj-docx-3-cre-10.1177_02692155231202855 - Supplemental material for Group-based cardiac telerehabilitation interventions and health outcomes in coronary patients: A scoping review Click here for additional data file. Supplemental material, sj-docx-3-cre-10.1177_02692155231202855 for Group-based cardiac telerehabilitation interventions and health outcomes in coronary patients: A scoping review by Renuka J Jacobsson, Anne Oikarinen, Jenni Krogell and Päivi Kankkunen in Clinical Rehabilitation

## Appendix 1

See Table A1.

**Table A1.** Summary of included studies.

Primary author (year) Study location	Study population and sample	Aim	Design/Method	Intervention type	Intervention setting	Outcome type	Follow-up	Health outcome(s)
Avila^18^ (2018) Belgium	Coronary artery disease patients *N* = 90	Evaluate the added benefit of a home-based cardiac rehabilitation program with telemonitoring guidance on physical fitness	Randomized controlled trial.	Home-based exercise intervention.	12 weeks. Telemonitoring guidance consisting of weekly emails or phone calls.	Physical fitness with pVO2 and oxygen uptake.	0-and 12-week	The increase in VO2 P was higher. Cardiac rehabilitation program results in further improvement of physical fitness.
Batalik^19^ (2021) Czech Republic	Coronary artery disease patients *N* = 56	Investigating a 1-year effect of a randomized controlled study using Cardiac Rehabilitation through the Global Position System compared to outpatient cardiac rehabilitation.	Randomized controlled trial.	Home-based cardiac rehabilitation program with regular physical exercise in the patient's home environment.	12 weeks. Two mandatory training sessions initiated home-based cardiac rehabilitation at the clinic under a physiotherapist's guidance and a cardiologist's supervision. During the pilot sessions, the patients were instructed how to exercise (load time, intensity) and were lent the HR Polar M430 wrist monitor.	pVO2 parameter, anthropometric parameters (a standard 12-lead electrocardiogram, gas exchange, and blood pressure), deaths and hospitalization. Quality of life: SF-36.	0-, 12-week and 1-year	Satisfactory long-term effects in pVO2, exercise performance, and perceived general health in CAD patients with low to moderate cardiovascular risk.
Bravo-Escobar^20^ (2017) Spain	Coronary artery disease patients *N* = 28	The objective of the study was to analyze the effectiveness and safety of a home-based cardiac rehabilitation program of mixed surveillance in patients with ischemic cardiopathology at moderate cardiovascular risk.	Randomized controlled trial.	home-based cardiac rehabilitation group.	Supervised physical exercise session once a week. Exercised at home following a walking program for 1 h in duration at 70% of the reserve heart rate following the Karvonen formula during the first month and 80% during the second, which was monitored with a remote electrocardiographic monitoring device NUUBO®	Anthropometric measurements (BMI, body weight, circumstance), blood pressure, exercise capacity (maximum heart rate) and laboratory parameters (total cholesterol, HDL cholesterol, LDL cholesterol, triglycerides, blood glucose and glycated hemoglobin).	0-and 8-week	Quality of life score was better in the control group. No significant differences in exercise time and recovery rate.
Brewer^38^ (2023) USA	Coronary patients with ≥1 lifestyle risk target *N* = 30	The aim was to assess the feasibility and acceptability of an intervention program, and changes in cardiovascular health behaviors, biometrics, knowledge and psychosocial factors.	Mixed method analysis.	A community informed virtual cardiac rehabilitation program.	8-week health education program using a virtual platform. Weekly peer support groups, lecture sessions, virtual fitness center, grocery store and restaurant tours.	Feasibility with recruitment adherence to intervention. Acceptability with focus group interviews. Health behavior, BMI, BP, cholesterol, LDL- C. Physical activity, health knowledge, psychosocial factors, quality of life and optimism questionnaires.	0- and 8-week	The program had high acceptability. Cardiovascular health behaviors and biometrics had clinically relevant trends, although not statistically significant.
Brewer^21^ (2017) USA	Coronary heart failure patients *N* = 8	The aim was to assess the feasibility and acceptability of a virtual web platform - based cardiac rehabilitation program as an extension to medical based cardiac rehabilitation.	Pilot study, A mixed-method approach.	Patients participated 12-week health education program.	Interactive, group virtual ‘field trips’ and session series delivered by expert health professionals specializing in cardiac rehabilitation delivery on a secure virtual web platform platform.	The pre-intervention survey assessed participant sociodemographic, social support,17 digital health information access, and prior virtual web platform experience.	0-and 12-week	Overall, there were positive participant perceptions of the virtual web platform experience. More than 80% of participants reported that the program improved their health knowledge and helped them maintain better health habits.
Brouwers^33^ (2022) The Netherlands	Coronary artery disease patients *N* = 55	The aim of the current study was to evaluate the long-term effectiveness of the cardiac telerehabilitation intervention applied in the FIT@Home trial6 on PAL, physical fitness and quality of life (QoL) after 4 years of follow-up, as compared with center-based cardiac rehabilitation.	Randomized controlled trial.	Cardiac telerehabilitation applying home-based training.	12-week training with a heart rate monitor, web application, and weekly telephone call.	Physical fitness (pVO2 assessed on a cycle ergometer), PAL (assessed using tri-axial accelerometry and heart rate data), and QOL (assessed using the MacNew questionnaire).	0-, 12-month, 1- and 4-year	The 12-week program does not prevent physical fitness and PAL over 4 years follow-up.
Calvo-Lopez^39^ (2023) Spain	Ischemic heart disease patients *N* = 62	The aim was to develop a home-based rehabilitation program and evaluate its impact.	Cross- sectional study.	A home-based cardiac telerehabilitation program.	8-week program including 3 weekly exercise sessions and 2 weekly educational sessions.	Functional capacity, adherence to a healthy lifestyle, quality of life.	0-and 8-week	Positive results in improving maximal aerobic, weekly training volume, muscle strength, compliance with diet, and anxiety symptoms.
Dinesen^22^ (2019) Denmark	Coronary artery disease patients *N* = 14 Coronary artery disease patients partner N = 12	The aim of the study was to explore the experiences of cardiac patients and their partners of participating in the Teledialog Telerehabilitation Program.	Case study.	The Teledialog program.	12-week program. A digital rehabilitation plan and transmission of health data from patient's home to hospital and health care center, and an interactive web portal with information and training videos.	The initial interview was to gain an understanding of the patients’ disease and rehabilitation plan and the everyday life of the patient and his/her partner. After 12- weeks interviewed again investigating the experiences.	0-and 12- week	Digital platform was a useful tool, helped coordinating goals and creating overviews of partner's rehabilitation activities. It improved individuality, sense of autonomy, enhanced the relatedness of health care professionals and a sense of competence.
Duan^23^ (2018) China	Coronary artery disease patients *N* = 136	The study's aim was to evaluate the effect of an 8-week w-based intervention.	Randomized controlled trial.	Web- based intervention.	8- week program. Social-cognitive variables, risk perception, outcome expectancies, and goal setting, action plans and coping plans. Behavior-specific social support.	Health Behavior, healthy lifestyle indicators, Social-Cognitive Indicators as Internal Resources of Behavior Change.	0-and 8-week	The intervention's effect was seen in the improvement of quality of life.
Gibson^34^ (2023) Ireland	Cardiac patients *N* = 105	To examine the impact of an evidence-based, digital CR program on medical, lifestyle and psychosocial outcomes.	Observational study.	Web-based program delivered by an interdisciplinary team of healthcare professionals.	During the 12-week program, patients were provided with a Fitbit fitness tracker, a home blood pressure (BP) monitor and an interactive workbook and access to a bespoke web-based platform and were invited to attend weekly, online group-based supervised exercise sessions and educational workshops.	Diet, anthropometric measurements, physical activity levels, functional capacity, psychosocial health, quality of life, blood pressure, fasting lipids, glucose and glycosylated hemoglobin, and prescribed medications.	0-, 12-week and 6-month.	Patients met their goals in physical activity, low-density lipoprotein cholesterol and mean weight reduction and adherence to the diet. Anxiety and depression levels both reduced. Most improvements were sustained at a 6-month follow-up.
Giggins^36^ (2023) Ireland	Coronary artery disease patients N = 21	To test the ECME-CR platform and examine the efficacy and feasibility of a remote CR exercise program.	Pilot study.	Web-based exercise classes and program.	8-week program, participants took part in web-based exercise classes and used the ECME-CR platform during the intervention period.	The primary outcome measure was exercise, capacity, assessed using a 6-min walk test (6MWT). Secondary outcomes included grip strength, self-reported quality of life, heart rate, blood pressure, and body composition.	0- and 8-week.	The pilot trial did not show evidence of a significant positive effect for either of the remotely delivered programs.
Higgins^25^ (2017) Australia	Coronary heart failure patients *N* = 21	Develop and pilot a flexible online cardiac rehabilitation program based on self-management principles.	Pilot study.	The Help Yourself Online program.	Web-based, online modules covering health behaviors (healthy eating, physical activity, medication adherence, smoking cessation) and emotional management (depression, anxiety, anger), as well as a module on social support.	Broader implications of the data include the acceptability of the intervention, timing of intervention delivery, and patients’ desire for additional online community support.	2–3 week to 2-month	Patients believed the program would assist them in their self-management.
Hwang^26^ (2017) Australia	Coronary heart failure patients *N* = 53	Is a home-based telerehabilitation program conducted in small groups non-inferior to a traditional center-based program.	Randomized controlled trial.	Home-based telerehabilitation.	12 weeks. Online videoconferencing software program with real-time exercise and education intervention delivered into the participant's home twice weekly.	6-min walk distance, other functional measures, quality of life, patient satisfaction, program attendance rates and adverse events.	0-, 12- and 24-week	The primary outcome was a positive change in the 6-min walk distance, with a non-inferiority margin of 28 m.
Hwang^27^ (2017) Australia	Coronary heart failure patients *N* = 17	To describe patient experiences and perspectives of a group-based heart failure (HF) telerehabilitation program.	Randomized controlled trial, mixed method study.	Group-based telerehabilitation program.	Online video- conferencing delivered to the homes.	Self- report surveys and semi-structured interviews to measure patient experiences and perspectives	Following 12- week	Health benefits, access to care and social support.
Knudsen^28^ (2020) Denmark	Coronary heart failure patients *N* = 77	To evaluate patient activation and health literacy in tele-rehabilitation compared to hospital-based cardiac rehabilitation.	Pilot study.	Home- based cardiac rehabilitation intervention.	12 weeks of supervised exercise training, dietary advice, educational and psychosocial support sessions to improve patients’ cardiovascular risk profile and reduce the recurrence of cardiac events.	Patient Activation Measure before the intervention, at the end of the intervention and at follow-up six months after the intervention. the Health Literacy Questionnaire.	0-, 12-week and 6-month	Understanding health information significantly improved 6 months after intervention.
Lahtio^35^ (2023) Finland	Coronary artery disease patients *N* = 59	To examine the effectiveness of remote technology in cardiac rehabilitation on physical function, anthropometrics, and quality of life (QoL) compared with conventional rehabilitation.	Randomized controlled trial.	Conventional cardiac rehabilitation with added remote technology.	The 12 months of rehabilitation consisted of three 5-day in-rehabilitation periods at a rehabilitation center. Between these periods were two remote 6-month self-rehabilitation periods.	Outcome measurements included the 6-min walk test, body mass, BMI, waist circumference, and World Health Organization QoL-BREF questionnaire	0- and 12-week	Reduction in waist circumference and increase in self-assessed quality of life were greater (environmental factors: 0.5; *P* = .02).
Lin^24^ (2018) Taiwan	Coronary artery disease patients *N* = 17	The study assessed the feasibility and acceptability of internet-based cognitive-behavior group therapy program, described the patterns of use and measured change in risk factors.	Pilot study	Internet-based cognitive-behavior group therapy program.	8-week Internet-based cognitive-behavior group therapy program. The asynchronous platform also sustained the interactions among participants and psychotherapists through emails, homework uploading and feedback, discussion forums, and electronic bulletin boards. An online video conference system was adopted to conduct the online group therapy using webcam videos for all the participants.	Psychological and psychophysiological measurements. Internet usage behavior of participants. Hostility, anxiety, and depression, as well as heart rate and respiration rate.	0- and 8- week	The treatment effectiveness of Internet-based cognitive-behavioral group therapy was comparable with a face-to-face one in reducing anxiety, hostility, respiration rate, and in improving vasodilation but not depression compared to a waiting-list control.
Ma^29^ (2021) China	Coronary artery disease patients *N* = 335	To evaluate the long-term effect of a smartphone-facilitated home-based cardiac rehabilitation model.	Observational cohort study.	Home-based cardiac rehabilitation tailored by WeChat.	Monitoring and telecommunication via smartphone app (WeChat). Educational materials weekly and individualized exercise prescription monthly.	Major adverse cardiac events, safety, quality of life, and physical capacity.	during a 24–42-month period	The home-based cardiac rehabilitation group had a much lower incidence of major adverse cardiac events higher in Seattle Angina Questionnaire score, and better control of risk factors.
Peng^30^ (2018) China	Coronary heart failure patients *N* = 98	To examine the effect of our telehealth exercise training program on health outcomes.	Randomized controlled trial.	Home-based telehealth exercise training program.	8 weeks. 32 exercise training sessions, with regular telephone or instant messaging follow-ups and consultations.	Minnesota Living with Heart Failure Questionnaire, 6-min walking test, resting heart rate, Hospital Anxiety and Depression Scale, left ventricular ejection fraction, and the New York Heart Association (NYHA) classification.	0-, 8- and 16-week	Significant improvements were in quality of life and 6-min walk-test.
Spindler^31^ (2019) Denmark	Coronary artery disease, coronary heart failure and valve surgery patients *N* = 136	How telerehabilitation provided adequate support for lifestyle changes and self-care efforts.	Randomized controlled trial.	Telerehabilitation- program.	12 weeks. Teledialog toolbox containing technology for the Teledialog project (a blood pressure monitor, scales, heart rate monitor, and a digital step counter, as well as a tablet personal computer with a mobile network).	Questionnaires on motivation for lifestyle changes and self- care psychological distress, and quality of life.	3-, 6-, and 12-month	An initial increase in autonomous motivation, but this positive difference in motivation did not last over time.
Su^37^ (2023) China	Coronary artery patients *N* = 20	To explore the experiences of patients who participated in an eHealth rehabilitation program.	A descriptive qualitative design with semi-structured individual in-depth interviews.	A nurse-led eHealth cardiac rehabilitation.	A social cognitive theory-driven telerehabilitation design features individual assessment and goal-setting cycle, telemonitoring, online educational modules, and synchronous communication.	Patients’ experiences were dichotomized into positive (enablers) and negative experiences (barriers). The positive experiences focused on patients’ behavioral change and how the rehabilitation influenced cognitive and social aspects.	After a 12-week telerehabilitation.	Five themes emerged: promoted behavior change and mitigated emotional distress. Cognitive determinants and offered social support improved. High affordability, accessibility, reliability of the rehabilitation, and expressed psychological, contextual, and technical barriers.
Su & Yu^32^ (2021) China	Coronary artery disease patients *N* = 146	To evaluate the effects of a nurse-led eHealth cardiac rehabilitation system.	Randomized controlled trial.	A nurse-led eHealth cardiac rehabilitation.	The nurse provided feedback on the patients’ goal attainment and lifestyle modifications on a weekly basis in a small group format through the WeChat platform, thus also mobilizing peer influence.	Health behaviors, cardiac self-efficacy, anxiety and depression, health-related quality of life, risk parameters and unplanned use of care services.	0-, 6 and 12-week	The findings of this study demonstrate the effectiveness of the intervention in modifying behavioral risk factors and improving health-related quality of life.
