# Supplementary material for: Group-based cardiac telerehabilitation interventions and health outcomes in coronary patients: A scoping review
Source: Clin Rehabil. 2023 Sep 21;38(2):184–201. doi: 10.1177/02692155231202855 (PMC10725089; doi:10.1177/02692155231202855)
Supplement: sj-docx-1-cre-10.1177_02692155231202855 - Supplemental material for Group-based cardiac telerehabilitation interventions and health outcomes in coronary patients: A scoping review [file sj-docx-1-cre-10.1177_02692155231202855.docx]

Search update

Data from 1 August 2022 to 15 July 2023

First search

Data from 1 January 2017 to 15 August 2022

Records screened
(n = 241)

Records excluded
(n = 233)

Full-text articles assessed for eligibility
(n= 8)

Full-text articles excluded, with reasons
(n=2)

2 Wrong study population (only individual)

Records after duplicates removed
(n= 241)

Records identified through database searches
(n = 344)

Records identified through database searches
(n = 1,745)

## Screening

## Included

## Eligibility

## Identification

Additional records identified through other sources (n=0)
(n = 0)

Records after duplicates removed
(n = 1,234)

Records screened
(n = 1,234)

Records excluded
(n =1,197)

Full-text articles assessed for eligibility
(n=36)

Full-text articles excluded, with reasons
(n =20)

6 wrong study population (only individual)

9 wrong study design

5 full-text not available

Studies included in the scoping review
(n =22)

Figure 1. Flow diagram of extracted studies.
