# Supplementary material for: Group-based cardiac telerehabilitation interventions and health outcomes in coronary patients: A scoping review
Source: Clin Rehabil. 2023 Sep 21;38(2):184–201. doi: 10.1177/02692155231202855 (PMC10725089; doi:10.1177/02692155231202855)
Supplement: sj-docx-2-cre-10.1177_02692155231202855 - Supplemental material for Group-based cardiac telerehabilitation interventions and health outcomes in coronary patients: A scoping review [file sj-docx-2-cre-10.1177_02692155231202855.docx]

Table 1. Search terms and results.

| Database | Search terms | Results | Results from updated search |
| --- | --- | --- | --- |
| Cinahl | (cardiac or cardiovascular or coronary or heart) AND rehabilitation* AND (internet or "mobile application" or virtual or "mobile apps" or smartphone* or "smart phone" or telenurs* or telemedicine or ehealth) | n=204 | n=29 |
| Pubmed | (cardiac or cardiovascular or coronary or heart) AND rehabilitation* AND (internet or "mobile application" or virtual or "mobile apps" or smartphone* or "smart phone" or telenurs* or telemedicine or ehealth) | n=754 | n=25 |
| Psycinfo | (cardiac or cardiovascular or coronary or heart) AND rehabilitation* AND (internet or "mobile application" or virtual or "mobile apps" or smartphone* or "smart phone" or telenurs* or telemedicine or ehealth) | n=126 | n=15 |
| Scopus | (cardiac or cardiovascular or coronary or heart) AND rehabilitation* AND (internet or "mobile application" or virtual or "mobile apps" or smartphone* or "smart phone" or telenurs* or telemedicine or ehealth) | n=661 | n=215 |
