# Supplementary material for: Group-based cardiac telerehabilitation interventions and health outcomes in coronary patients: A scoping review
Source: Clin Rehabil. 2023 Sep 21;38(2):184–201. doi: 10.1177/02692155231202855 (PMC10725089; doi:10.1177/02692155231202855)
Supplement: sj-docx-3-cre-10.1177_02692155231202855 - Supplemental material for Group-based cardiac telerehabilitation interventions and health outcomes in coronary patients: A scoping review [file sj-docx-3-cre-10.1177_02692155231202855.docx]

Table 3. Summary of telerehabilitation devices and applications used in the included studies on group-based cardiac telerehabilitation.

| **Computer/tablet** | **Mobile connec-tion** | **Videoconfe**  **rencing** | **Chat** | **Telemoni-**  **toring** | **Virtual platform** | **Technology based feed- back/coaching** | **Study** |
| --- | --- | --- | --- | --- | --- | --- | --- |
| x | x |  |  | x |  | x | Avila^18^ (2018) |
| x | x |  |  |  |  |  | Batalik^19^ (2021) |
| x |  |  |  | x |  |  | Bravo- Escobar^20^ (2017) |
| x |  | x | x |  | x | x | Brewer^38^ (2023) |
| x |  | x | x |  | x | x | Brewer^21^ (2017) |
| x | x |  |  | x |  | x | Brouwers^33^ (2022) |
| x | x | x | x | x | x | x | Calvo- Lopez^39^ (2023) |
| x |  |  |  |  | x |  | Dinesen^22^ (2019) |
| X | x |  |  |  | x | x | Duan^23^ (2018) |
| X | x | x | x | x | x | x | Gibson^34^ |
| x | x | x |  | x |  | x | Giggins^36^ |
| x | x |  | x | x | x | x | Lahtio^35^ |
| x | x | x | x | x | x | x | Lin^24^ (2018) |
| x |  |  | x |  | x | x | Higgins^25^ (2017) |
| x |  | x | x |  |  | x | Hwang^26^ (2017) |
| x |  | x | x |  |  | x | Hwang^27^ (2017) |
| x | x |  |  | x | x | x | Knudsen^28^ (2020) |
| x | x |  | x | x |  | x | Ma^29^ (2021) |
| x |  | x | x | x | x | x | Peng^30^ (2018) |
| x |  |  | x | x | x | x | Spindler^31^ (2019) |
| x | x |  | x | x | x | x | Su^37^ (2022) |
| x |  |  | x |  | x | x | Su & Yu^32^ (2021) |
